# Memorial Resolution, University of Buffalo

**Published:** 1911-06

**Authors:** Matthew D. Mann, Eli H. Long

**Affiliations:** Dean; Secretary


### Memorial Resolution, University of Buffalo

At a meeting of the faculty of the medical department of the University held on May 8, the following was adopted:

The faculty of the medical department of the University of Buffalo wish to express their great sense of loss and their deep sorrow on the death of Carlton C. Frederick. Dr. Frederick was for many years a distinguished and very useful member of the faculty. As a teacher he possessed the respect and confidence of the students, and the high regard and friendship of his associates. His high professional attainments, his great learning and large experience made him an authority in his department and rendered his teaching of great value; while his uprightness of character and known championship of all that was right and noble in the profession made him a worthy example to those under him.

While we must meet his loss with courage and resignation, we can do it only with deep and lasting regret. To his family we extend our sincere sympathy.

(Signed) Matthew D. Mann, *Dean,*
Eli H. Long, *Secretary.*

